# Three-dimensional growth and biomechanical risk progression of abdominal aortic aneurysms under serial computed tomography assessment

**DOI:** 10.1038/s41598-023-36204-2

**Published:** 2023-06-07

**Authors:** Antti Siika, Marko Bogdanovic, Moritz Lindquist Liljeqvist, T. Christian Gasser, Rebecka Hultgren, Joy Roy

**Affiliations:** 1 Division of Vascular Surgery, Department of Molecular Medicine and Surgery, Karolinska Institutet, BioClinicum J8:20 Visionsgatan 4, 171 64 Solna, Stockholm, Sweden; 2 Department of Vascular Surgery, Karolinska University Hospital, Stockholm, Sweden; 3 KTH Solid Mechanics, Department of Engineering Mechanics, KTH Royal Institute of Technology, Stockholm, Sweden; 4 Faculty of Health Sciences, University of Southern Denmark, Odense, Denmark

**Keywords:** Health care, Cardiovascular diseases

## Abstract

Growth of abdominal aortic aneurysms (AAAs) is often described as erratic and discontinuous. This study aimed at describing growth patterns of AAAs with respect to maximal aneurysm diameter (Dmax) and aneurysm volume, and to characterize changes in the intraluminal thrombus (ILT) and biomechanical indices as AAAs grow. 384 computed tomography angiographies (CTAs) from 100 patients (mean age 70.0, standard deviation, SD = 8.5 years, 22 women), who had undergone at least three CTAs, were included. The mean follow-up was 5.2 (SD = 2.5) years. Growth of Dmax was 2.64 mm/year (SD = 1.18), volume 13.73 cm^3^/year (SD = 10.24) and PWS 7.3 kPa/year (SD = 4.95). For Dmax and volume, individual patients exhibited linear growth in 87% and 77% of cases. In the tertile of patients with the slowest Dmax-growth (< 2.1 mm/year), only 67% belonged to the slowest tertile for volume-growth, and 52% and 55% to the lowest tertile of PWS- and PWRI-increase, respectively. The ILT-ratio (ILT-volume/aneurysm volume) increased with time (2.6%/year, *p* < 0.001), but when adjusted for volume, the ILT-ratio was inversely associated with biomechanical stress. In contrast to the notion that AAAs grow in an erratic fashion most AAAs displayed continuous and linear growth. Considering only change in Dmax, however, fails to capture the biomechanical risk progression, and parameters such as volume and the ILT-ratio need to be considered.

## Introduction

Abdominal aortic aneurysms (AAAs) are irreversible dilatations of the abdominal aorta that are associated with a risk of rupture^1^. Rupture is often a fatal event^2^, and currently the only effective treatment is elective surgery prior to rupture, by either open surgical repair or endovascular repair^1,3^. As AAAs are only effectively treated before rupture, but are in general asymptomatic, they are either found through incidental detection on radiological examinations, or specifically designed screening programs. Once an AAA is found, patients are put under surveillance, with primarily ultrasound, and followed until the AAA reaches the surgical-threshold diameter^1,3^.

Diameter growth of aneurysms has been described as discontinuous, erratic and nonlinear^4–7^. The inter-observer variability of maximal aneurysm diameter (Dmax) measurements, between both computed tomography (CT) and ultrasonography are significant^8–10^. Even for a CT of a single patient with an AAA, many ways of measuring the maximal aneurysm diameter have been described^8^. The fastest diameter growth in an AAA does not always occur at the Dmax location^11^, and it has been suggested that volume growth is a more sensitive marker of aneurysm disease progression^12,13^. Semi-automatic measurements, where a diameter is computationally measured from a segmented aneurysm may be more accurate compared to manual diameter readings, and may contribute to improved growth prediction of AAAs^14^.

Biomechanical analysis has shown potential to improve prediction of both AAA rupture and growth. Peak wall stress (PWS) is higher in ruptured AAAs^15,16^, and peak wall rupture index (PWRI), is increased in AAAs prior to rupture^17^. Further, PWRI correlates to aneurysm volume-growth^13^. However, only small studies have reported on the development over time of these biomechanical parameters^18,19^.

The primary aim was to investigate the growth pattern of AAAs with respect to semi-automatic Dmax and volume assessment in a cohort of patients that had undergone at least three CT angiograms (CTAs). The secondary aim was to characterize the changes in intraluminal thrombus (ILT) and biomechanical properties (PWS and PWRI) in relation to AAA growth.

## Methods

### Study cohort

Patients who presented with intact AAA (ICD code I71.4) to the Department of Vascular Surgery at Karolinska University Hospital between the years 2012–2013 were screened for inclusion. Patients were included if they had undergone ≥ 3 CTAs, that were at least 3 months apart, at any point. At our centre patients with AAAs are in general surveyed with ultrasound, and many of the CTs included may have been performed for indications other than AAA. Patients with mycotic, infectious or thoracoabdominal AAAs were excluded. CTAs were extracted from the hospital picture archiving and communications systems. Patient characteristics including age, sex and smoking status were collected from the electronic medical records.

### Geometric characterization and finite element analysis

For 3D-segmentation and Finite element analysis, A4Clinics (VASCOPS GmbH, Graz, Austria) was used. The program is commercially available, and the methods are detailed elsewhere^20,21^.

In short, the analysis is initiated by segmentation of the AAA, including lumen, ILT and vessel wall. The segmentation is semi-automatic, and based on deformable balloon models which generate a hexahedral dominated mesh for the finite element analysis^20^. The model is given constitutive properties that represent the stress and strain relation of the tissue. These are assigned based on data from ex-vivo biaxial tensile testing. The wall and the thrombus were both model as hyperelastic, isotropic and incompressible^22,23^. The wall strength of the AAA is inhomogeneous and estimated based on geometric characteristics (relative expansion of the aorta, the thickness of overlying thrombus), and can be adjusted with patient characteristics (sex, age and heredity for rupture, long-term blood pressure)^24^. The model is fixed at the boundaries, the renal arteries and the aortic bifurcation, and no interaction with the surrounding tissue was considered.

All patients were simulated with the same patient characteristics (65 years old, male, blood pressure 140/80 mmHg and no heredity for rupture). These patient characteristics were used to neutralize their effect on the wall strength model and thereby study the effects of changing aneurysm morphology on the biomechanical parameters. For all geometric and biomechanical parameters, the aneurysm was considered between the lowest renal artery (excluding polar arteries) and the aortic bifurcation. For each segmented CTA, the software outputs the following parameters that are considered in this paper: maximal aneurysm diameter (Dmax), total aneurysm volume, ILT volume, lumen volume, peak wall stress (PWS) and peak wall rupture index (PWRI). PWS denotes the maximum von Mises stress at any point in the AAA, and PWRI is the maximum ratio of wall stress to wall strength^25^. The maximal diameter is measured perpendicular to the vessel centreline and corresponds to the *maximal diameter in any direction* within the cross-section. ILT ratio is defined as ILT volume / total aneurysm volume.

CTA images with a slice thickness of > 5 mm were excluded. If slice thickness was > 3 mm (n = 25), images were resampled isotropically with b-splines using the 3D slicer software (version 4.11.0–2020-07–09)^26^ to allow for more detailed reconstruction.

### Statistical analysis

All statistical analyses were performed using R version 4.04 (R Foundation for Statistical Computing, Vienna, Austria)^27^. Statistical significance was defined as *p* < 0.05.

To account for repeated observations for each patient, data were modelled using mixed-effects models fitted with the R packages *lmerTest*^28^. Mixed-effects models allow for the specification of patient-level random slopes and intercepts. For all models in this paper random intercepts and slopes are specified. The influence of patient characteristics on the time-dependent change of the analysed geometric and biomechanical parameters are modelled with an interaction term between time and the specified patient characteristic. The presented estimates represent the marginal effects and are calculated using the *margins* package^29^, and the corresponding p-value denotes the statistical significance of the time-interaction variable. Patient-level growth rates for Dmax, aneurysm volume, lumen volume and ILT volume, as well as relative growth rates for Dmax and volume represent patient-level conditional estimates from the corresponding mixed-effects model.

To evaluate the fit of a simple time-dependent linear model to the data, each patient was fitted with a linear regression with the morphological or biomechanical variable as the outcome and time as the independent variable. An r-squared value above 0.90 was considered as appropriate to describe the growth of the individual patient as linear, this definition was also used by others^30^. Difference in correlation coefficients was tested with the Dunn and Clark’s test, as implemented in the *cocor*-package.

### Ethical considerations

The study was approved by the Regional Ethical Review Board in Stockholm and conformed to the principles outlined in the Declaration of Helsinki. Informed consent was not considered necessary. Individual patient data will not be made publicly available since it was not part of the Ethical approval for this study.

## Results

### Patient cohort

For all 100 included patients and 384 CTAs, FEA and geometric characterization was performed. Patients had a mean age of 70.0 (SD = 8.5) years, were mostly male (78%) and a majority (87%) had a history of smoking. Fifty-two patients had 3 CTs, 26 had 4 CTs and 22 patients had 5 or more CTs included. The mean total follow-up (time between first and last included CT) was 5.2 (SD = 2.5) years, and the mean time between CTs was 2.7 (SD = 1.5) years. At baseline, Dmax was 43.9 (SD = 6.8) mm and PWS was 169 (SD = 44) kPa. The mean growth rate of the Dmax in the cohort was 2.64 (SD = 1.18) mm / year, mean aneurysm volume growth was 14.3 (SD = 10.2) cm3 / year and the mean PWS increased 7.4 (SD = 5.0) kPa/year (Table 1).

**Table 1:** Summary of baseline patient characteristics.

Patient characteristics (n = 100)		
Age at baseline -yrs	70.0 (8.5)	
Sex = Male	78 (78%)	
Current smoker	36 (36%), Missing: 1	
Ever smoker	86 (87%), Missing: 1	
Height -cm	174.0 (8.8), Missing: 5	
Weight -kg	82.6 (16.6), Missing: 6	
BSA -m^2^	1.97 (0.22), Missing: 6	
BMI -kg/m^2^	27.1 (4.5), Missing: 6	

CTAs (n = 384)		
Median No. of CTA s per patient –n	3.0 (3.0–4.0)	
Mean time between CTAs -years	2.7 (1.5)	
Mean total follow-up time -years	5.2 (2.5)	

Measurements (n = 100)	**Baseline** Mean (sd)	**Crude growth** **rate–** estimate (/year) (sd) ^†^
Dmax -mm	43.9 (6.8)	2.64 (1.18)
Aneurysm volume -cm^3^	94 (33)	14.28 (10.24)
Lumen volume -cm^3^	53 (19)	5.05 (6.04)
ILT volume -cm^3^	23 (20)	8.00 (7.24)
Peak wall stress -kPa	169 (44)	7.39 (4.95)
Peak wall rupture index -%	29.6 (8.1)	2.38 (2.14)

BSA = body surface area, BMI = body mass index, CTA = computed tomography angiography, Dmax = maximal aneurysm diameter, ILT = intraluminal thrombus. Values denote n (%), mean (standard deviation) or median (interquartile range). ^†^ Estimates refer to estimates from mixed effects models, where the variable is a function of time with random intercepts and slopes. Standard deviation refers to the variability in the random slopes.

**Figure 1:**
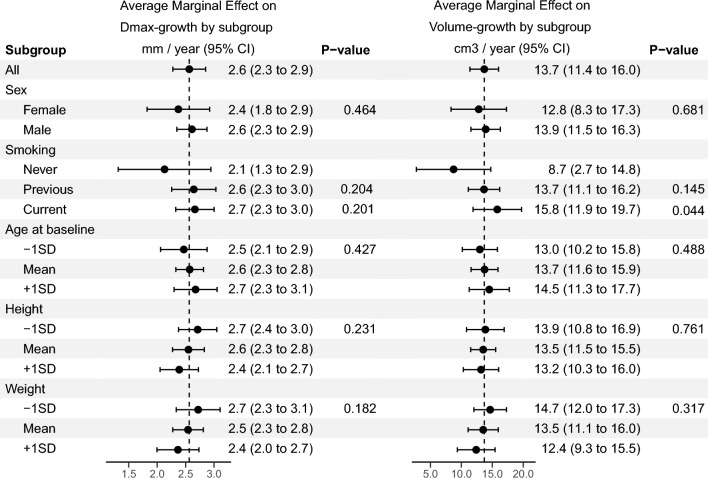
Subgroup analysis of AAA growth over time for Dmax (maximal aneurysm diameter) and aneurysm volume. Average marginal effect and 95% confidence interval around the estimate refer to estimate growth for patients belonging to the subcategory. *P*-value refers to the significance of the interaction term between time and the subgroup.

Women and men displayed similar growth rates for Dmax (2.4, 95% CI 1.8–2.9 mm/year vs 2.6, 2.3–2.9 mm/year, *p* = 0.46) and aneurysm volume (12.8, 8.3–17.3 cm3/year vs 13.9, 11.5–16.3 cm3/year, *p* = 0.68) (Fig. 1). Current smokers had a significantly higher volume growth rate compared to never smokers, (*p* < 0.044). Increasing age at baseline was associated with a slower increase in PWRI (*p* = 0.036). Other patient characteristics had no significant interaction with time for Dmax and Aneurysm volume (Fig. 1), or, PWS and PWRI (Supplementary Fig. 1).

**Figure 2:**
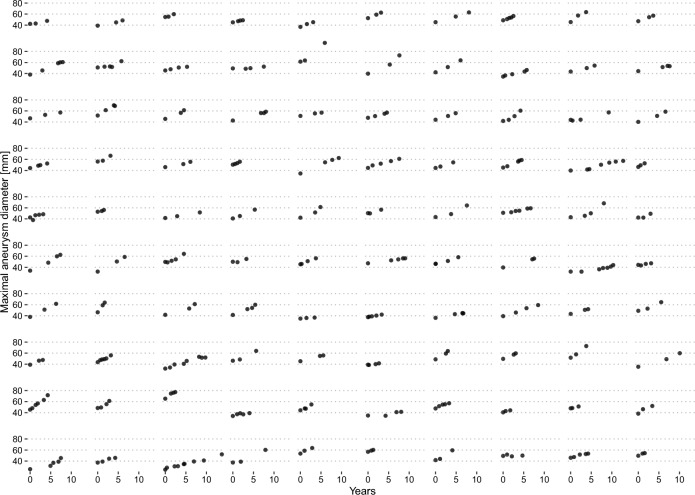
Maximum aneurysm diameter over time for all patients that are included in the study. A single plot represents one patient, and a dot represents one CTA examination. Y axis denotes the maximum aneurysm diameter (mm), and the x-axis time (in years) from inclusion into the study.

Dmax-growth appeared qualitatively continous and mostly linear (Fig. 2). Individual linear-regression models fitted to each patient revealed an excellent fit (r-squared > 0.90) for 87% of patients, and a mean r-squared of 0.94 ± 0.12. A simple linear-time model for aneurysm volume had an excellent fit in 77% of cases. Growth curves for aneurysm volume over time for the included patients in the study are presented in Supplementary Fig. 2. For Lumen volume, ILT-volume, PWS and PWRI the r-squared value was above 0.90 in 39%, 61%, 38% and 35% of cases respectively (Table 2). Despite this, the mean r-squared values for all fits was above 0.65.

**Table 2:** Goodness of fit for individual linear regression models fitted to each patient.

	R-squared		
	Mean	SD	> .90 (n)^†^
Dmax	0.94	0.12	87
Aneurysm volume	0.91	0.16	77
Lumen volume	0.72	0.29	39
ILT-volume	0.81	0.26	60
Peak wall stress	0.66	0.33	38
Peak wall rupture index	0.72	0.30	35

SD = standard deviation, Dmax = maximal aneurysm diameter, ILT = intraluminal thrombus. ^**†**^The number of patients where the r-squared value for the model exceeds 0.90.

For twelve selected patients, Dmax measurements collected from the electronic medical records, measured with various modalities, are presented in Supplementary Fig. 3, together with the semi-automatic maximal diameter measurements. There is substantial variation, depending on which modality is used for measuring. In some cases, such as for the fifth patient, the clinical CT diameter appears stagnant, until it suddenly increases, in contrast to the semi – automatic diameter which increases continuously.

### Comparison of maximal aneurysm diameter, aneurysm volume and peak wall stress growth rates

Patients were divided into groups based on tertiles of Dmax-growth (less than 2.11 mm/year, 2.11 mm/year–3.04 mm/year and more than 3.04 mm/year), Volume, PWS and PWRI (Table 3). Dmax-growth tertiles were not directly related to the corresponding aneurysm volume, PWS or PWRI growth distributions. For patients in the lowest Dmax-growth tertile, only 67% belong to the lowest tertile of aneurysm volume growth, and 52% belong to the lowest PWS-increase tertile and 55% to the lowest tertile of PWRI-increase (Table 3).

**Table 3:** Patients categorized according to growth in tertiles (slow, intermediate, and fast) by Maximal Aneurysm Diameter (Dmax), volume and peak wall stress (PWS), peak wall rupture index (PWRI).

	Dmax-growth (mm/year)		
	Slow (< 2.11), N = 33^1^	Intermediate (2.11–3.03), N = 34^1^	Fast (> 3.03), N = 33^1^
**Volume-growth (cm3/year)**			
Slow (< 9.6)	22 (67%)	11 (32%)	0 (0%)
Intermediate (9.6–16.74)	10 (30%)	14 (41%)	10 (30%)
Fast (> 16.74)	1 (3.0%)	9 (26%)	23 (70%)
**PWS-growth (kPa/year)**			
Slow (< 5.38)	17 (52%)	10 (29%)	6 (18%)
Intermediate (5.38–8.48)	10 (30%)	15 (44%)	9 (27%)
Fast (> 8.48)	6 (18%)	9 (26%)	18 (55%)
**PWRI-growth (ratio/year)**			
Slow (< 0.01)	18 (55%)	11 (32%)	4 (12%)
Intermediate (0.01–0.03)	11 (33%)	14 (41%)	9 (27%)
Fast (> 0.03)	4 (12%)	9 (26%)	20 (61%)

^1^n (%).

### Lumen and ILT volume changes

**Figure 3:**
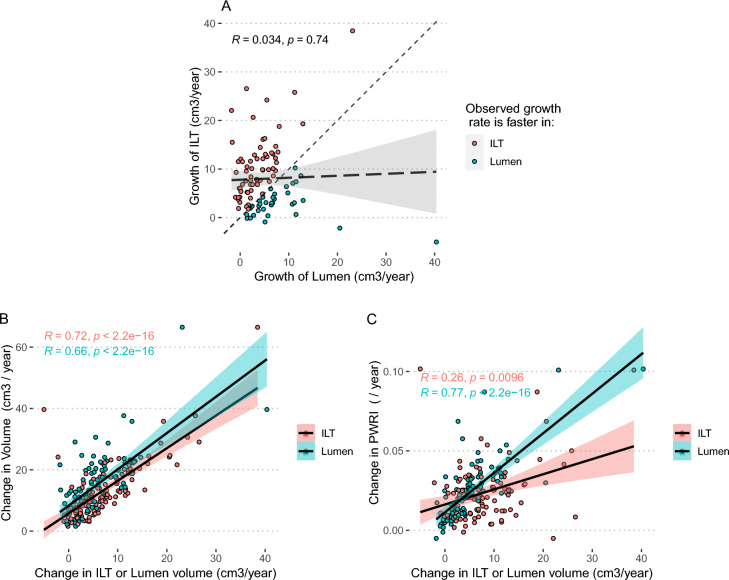
Scatter and correlation plots. (**A**) Change of intraluminal thrombus (ILT) volume plotted against change of lumen volume, (**B**) Change of Aneurysm Volume plotted against change of ILT volume (red dots), or lumen volume (blue dots). (**C**) Change in PWRI plotted against change of ILT volume (red dots), or lumen volume (blue dots). Correlation coefficients and respective *p*-values are shown in the figure.

In the majority of patients ILT-volume increased faster than lumen volume, and consequently the ILT-ratio (ILT-volume / aneurysm volume) in aneurysms increased over time (2.63%, 2.13–3.14% / year, *p* < 0.01) (Supplementary table 1). There was, however, no correlation between the growth of ILT and the growth of the lumen (r = 0.034, *p* = 0.74) (Fig. 3A). Both ILT and lumen volume change correlated with change in volume (r = 0.72, *p* < 0.001, and r = 0.66, *p* < 0.001), there was no evidence of difference in the correlations (*p* = 0.39) (Fig. 3B). Change in lumen volume correlated significantly stronger with change in PWRI (r = 0.77, *p* < 0.001) compared to change in ILT volume (r = 0.26, *p* = 0.001, p for difference in correlations coefficients < 0.001) (Fig. 3C).

**Figure 4:**
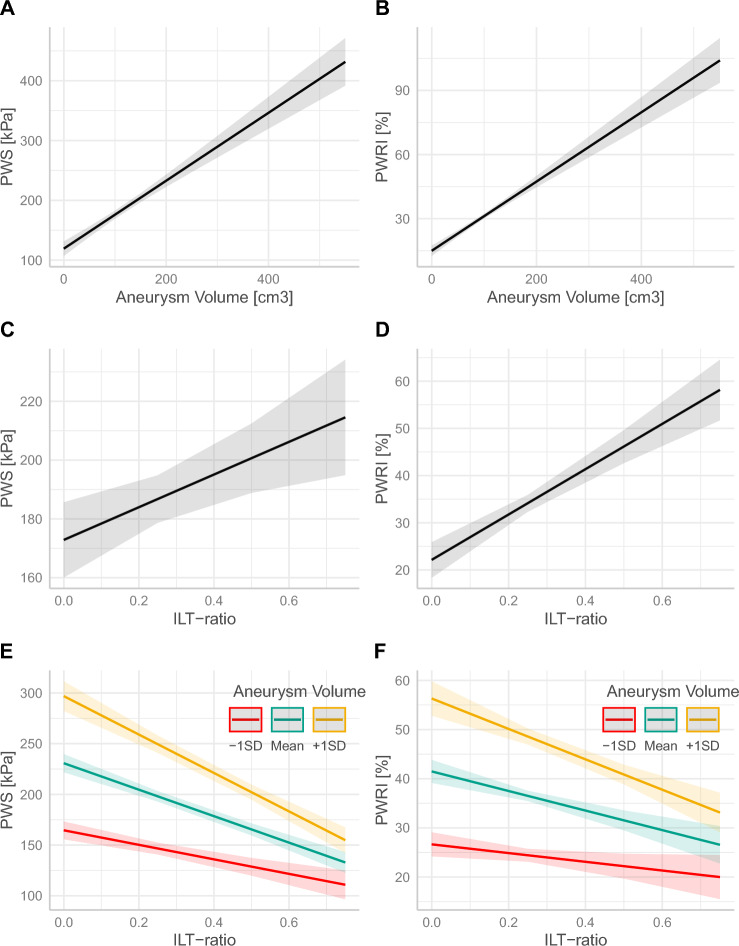
Relation between biomechanical indices (PWS and PWRI) and aneurysm volume (**A**, **B**), ILT ratio (**C**, **D**) and ILT-ratio adjusted for aneurysm volume and their interaction (**E**, **F**) Coloured lines indicate the relation of biomechanical stress to ILT-ratio at different levels (-1sd, mean and +1sd) of aneurysm volume.

Aneurysm volume and ILT ratio were both associated with increasing PWS and PWRI, when modelling them separately (Fig. 4A–D). However, when introducing an interaction term between aneurysm volume and ILT-ratio, PWS and PWRI still increase for any given aneurysm volume, but they instead decreased with increasing ILT-ratio (Fig. 4E–F and Supplementary table 2).

## Discussion

The present study shows that Dmax and volume growth, generally, do not appear to be erratic or discontinuous. Dmax-growth is, in many cases, adequately described as linear. A slow Dmax growth, however, does not harmonize with a sometimes more rapid volume growth and biomechanical increase. The proportion of ILT increased with time but was related to lower biomechanical stress.

### Maximal aneurysm diameter and volume growth

Previous studies have described that AAA growth is erratic and discontinuous^4–7^. Contrary to this notion, where aneurysm growth largely is considered as unpredictable, our results instead indicate that aneurysms mostly grow continuously, and for many patients, growth patterns can adequately be described by a linear model. This is in line with recently published results by Olson et al., who reported from the Non-Invasive Treatment of Abdominal Aortic Aneurysm Clinical Trial (N-TA^3^CT) that most patients included therein displayed continuous and linear Dmax-growth patterns^30^. The follow up of patients was between 18 and 30 months in the N-TA^3^CT trial, in this study we show similar growth patterns but importantly also during longer follow up time (mean 5.2 years). In the current study, volume growth could also, in most patients, be adequately described by a linear model. There may be several explanations for these previous observations of erratic aneurysm growth. Studies that are based on manual measurements from a clinical setting may suffer from certain biases and methodological errors. Semi-automatic diameter measurements for instance are not affected by rounding or so-called ‘terminal digit preference’, which is reported in standard diameter measurements^31^. Further, in the clinical setting, especially regarding ultrasound, the observer may be unaware of the exact location and method of the previous measurement. In this study, the AAAs were segmented semi-automatically and measurements were extracted from the resulting 3D model. This may overcome many of the potential sources of bias in manual measurement and be more representative of the true biological growth.

While the growth of most patients could be described as linear, for Dmax and volume growth 13% and 23% of patients respectively did not confer to the linear model. The current surveillance strategy with increasing frequency of measurements as the aneurysm grows^32^, is motivated by the notion of non-linear, exponential, growth. Further work is needed to predict the growth pattern of individual aneurysms, as those growing exponentially should be monitored with increasing frequency and the surveillance intervals of those growing linearly could be extended. The continuous nature of AAA growth described in this study, however, reveals potential for optimized growth prediction from taking more than one observation into account.

Patients with slow Dmax-growth rates in some cases exhibit higher aneurysm volume and PWS/PWRI change rates. Conversely, some patients with a high Dmax-growth rate exhibit lower aneurysm volume and biomechanical change rates. These findings support previous notions that there is a clear difference between surveillance of Dmax and aneurysm volume^33^. Also, aneurysm biomechanics may evolve independent from aneurysm diameter change. This points to potentially added value of 3D surveillance of AAAs, perhaps particularly in the research setting to for instance interpret the effect of pharmacological therapy. Further studies are, however, required to determine the clinical role of AAA volume or biomechanical parameters in surveillance.

### The role of the ILT

There are conflicting reports in the literature regarding the role of the ILT in AAA growth and rupture. The wall of the aneurysm directly affected by the ILT is more degraded, thinner, and weaker^34,35^. The ILT, however, seems to cushion stress from the wall^36^. The proportion of the ILT in an AAA has been associated with AAA growth^37,38^, especially a thick ILT seems to preclude growth^39^, and the growth of the ILT has been implicated in rupture^40^. Conversely, ruptured AAAs appear to have less thrombus^41,42^.

It is suggested that an interplay of the different effects of the ILT lead to an U-shaped association between ILT thickness and aneurysm growth, where a thin ILT does not provide a cushioning effect, while a thick ILT has a greater cushioning effect but a larger effect on wall degradation and therefore also leads to AAA growth^39^. The present study observes that the proportion of ILT (ILT-ratio) increases as aneurysms grow, but when adjusting for aneurysm volume, ILT-ratio is associated with decreased biomechanical stress. Thus, given an aneurysm of certain size, biomechanical rupture risk appears to be inversely related to the proportion of thrombus. Together with the literature, these results point to a two-fold role of the ILT, where the ILT potentiates growth of AAAs, and the continuous deterioration of the aneurysm wall, whereas it provides biomechanical support. The positive association between the amount of ILT and the size of the AAAs, may in some cases cause confounding. Further, in the case of rupture of the ILT, biomechanical stress may be transmitted through the thrombus to the underlying wall which likely negates the protective effects of ILT. Overall, the net effect of the ILT on growth and rupture likely depends on a number of factors.

### Limitations

The nature of this data is retrospective, and there is selection bias in the AAAs that are included. In addition, to be able to study CTA-based growth rates we included only patients that had undergone a minimum of three examinations. However, the overall growth rate of AAAs was 2.6 mm/y, and this is comparable with previously reported growth rates^43,44^. For some patients, it is obvious that they were deemed as unfit for elective repair, as there are several aneurysms that grow beyond the current operative threshold recommendations and this patient group may represent a comparatively more comorbid patient-group compared to AAA patients in general.

## Conclusions

Dmax and volume growth, generally, do not appear to be erratic or discontinuous. Dmax-growth can in most cases adequately be described as linear. Patients with slow Dmax growth do, however, sometimes display relatively more rapid volume growth and PWS increase. The proportion of ILT increased with time but was related to lower biomechanical stress.

## Supplementary Information

Supplementary Information.

## Data Availability

The data comes from patients, where informed consent has been waived by the Stockholm Ethical Review Board. Individual patient data will not be made publicly available since it was not part of the Ethical approval for this study. Data is available from Antti Siika (antti.siika@ki.se) or Joy Roy (joy.roy@ki.se), upon reasonable request with appropriate ethical permission.
