# Supplementary material for: Three-dimensional growth and biomechanical risk progression of abdominal aortic aneurysms under serial computed tomography assessment
Source: Sci Rep. 2023 Jun 7;13:9283. doi: 10.1038/s41598-023-36204-2 (PMC10247735; doi:10.1038/s41598-023-36204-2)
Supplement: Supplementary file 1 — Supplementary Information. [file 41598_2023_36204_MOESM1_ESM.pdf]

Supplementary Material

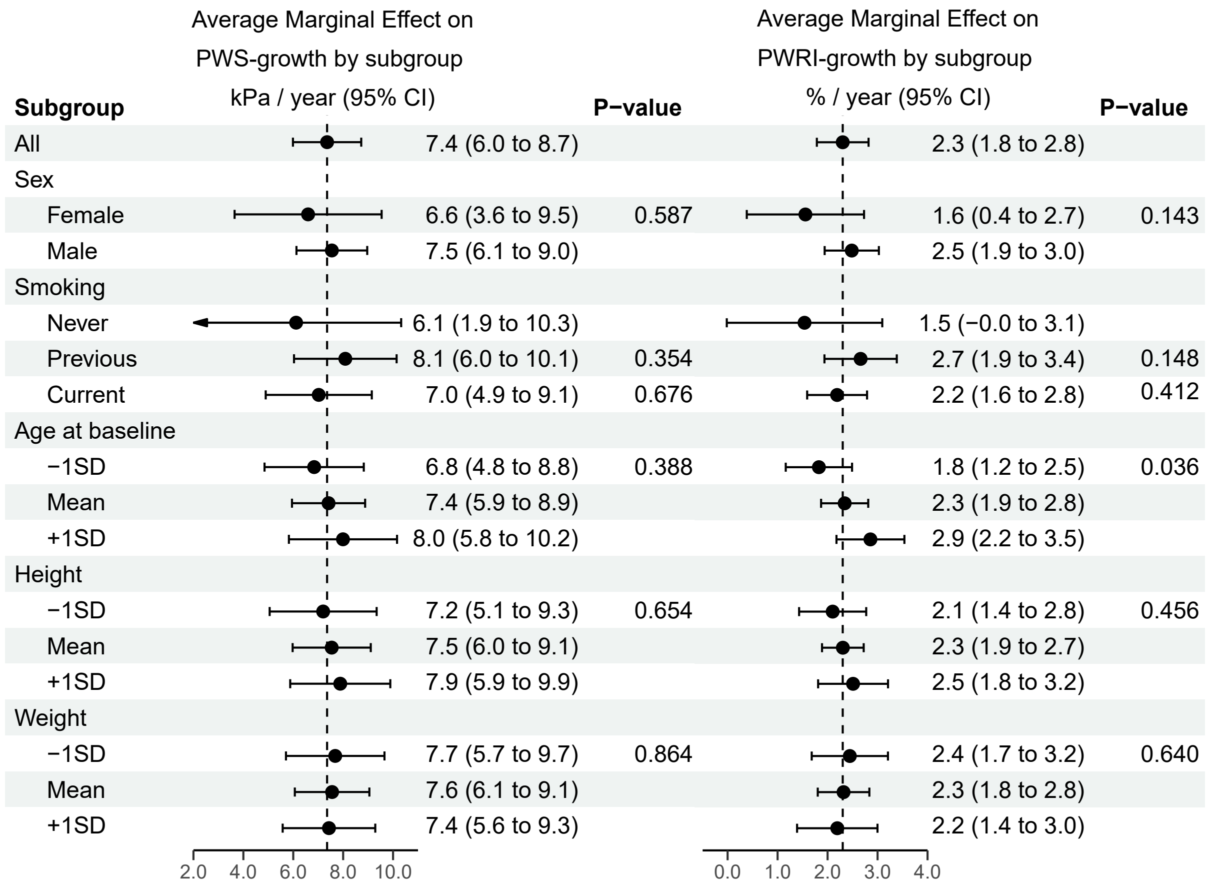

Supplementary Figure 1. Growth rates of PWS and PWRI by patient characteristics.

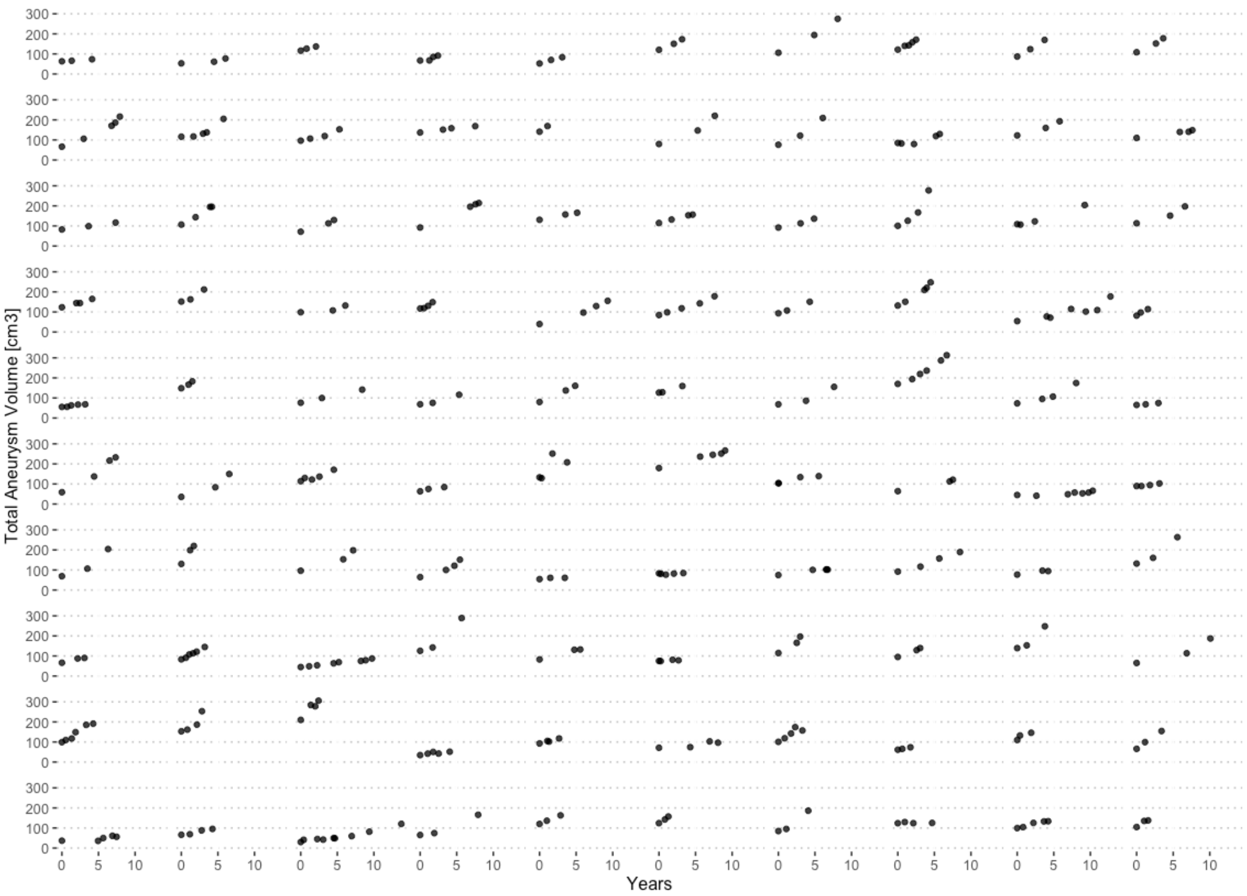

Supplementary Figure 2. Total aneurysm volume over time. A single plot represents one patient, and a dot represents one CTA examination. Y axis denotes the maximum aneurysm volume (cm3), and the x-axis time (in years) from inclusion into the study.

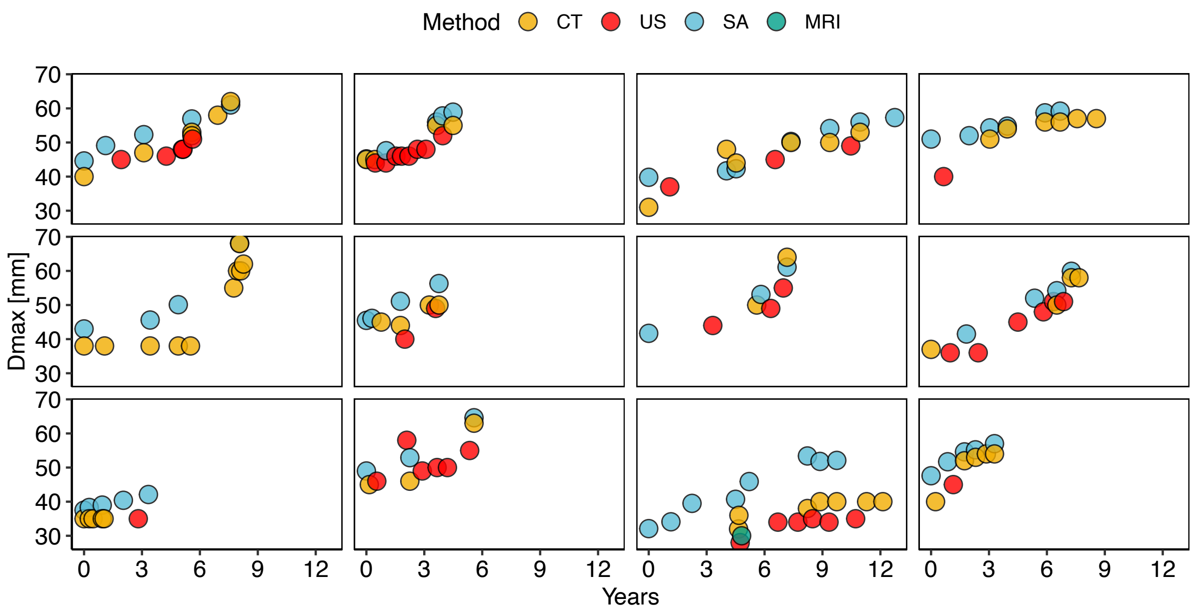

Supplementary Figure 3. Diameter measurements for twelve patients over time. Colors indicate CT (computed tomography), US (ultrasound), SA (semi-automatic), and MRI (magnetic resonance imaging).

Supplementary Table 1. Mixed effects model of ILT-ratio over time.

	ILT ratio (%)		
Predictors	*Estimates*	*CI*	*p*
(Intercept)	22.25	19.39 – 25.11	**<0.001**
ILT-ratio % /year	2.63	2.13 – 3.14	**<0.001**
Observations	384		
Marginal R^2 / Conditional R^2	0.166 / 0.919		

Supplementary table 2. Regression model of peak wall stress and peak wall rupture index as estimated by ILT ratio and aneurysm volume

	**Peak Wall Stress (kPa)**			**Peak Wall Rupture Index (%)**		
Predictors	*Estimates*	*95 % CI*	*p*	*Estimates*	*95% CI*	*p*
(Intercept)	83.58	67.44 – 99.72	**<0.01**	9.47	5.80 – 13.15	**<0.01**
ILT-ratio (%)	33.19	-10.84 – 77.23	0.14	6.92	-4.08 – 17.93	0.22
Aneurysm Volume (cm3)	1.16	1.03 – 1.29	**<0.01**	0.25	0.22 – 0.29	**<0.01**
IIT-ratio * Aneurysm volume	-1.25	-1.55 – -0.96	**<0.01**	-0.19	-0.26 – -0.12	**<0.01**
Observations	384			384		
Marginal R^2 / Conditional R^2	0.609 / 0.893			0.216 / 0.961		
